# Development of a handoff continuity score to improve pediatric ICU physician schedule design for enhanced physician and patient continuity

**DOI:** 10.1186/cc10504

**Published:** 2011-10-21

**Authors:** Hannah K Smalley, Pinar Keskinocak, Atul Vats

**Affiliations:** 1 Georgia Institute of Technology, 765 Ferst Drive NW, Atlanta, GA 30332, USA; 2 Children's Healthcare of Atlanta and Emory University School of Medicine, 1405 Clifton Road NE, Atlanta, GA 30322, USA

## Abstract

**Introduction:**

Few studies investigate the benefits of familiarity or continuity during physician-to-physician handoff of inpatients. Factors such as how recently physicians (MDs) have worked and successive days caring for patients increase continuity, and thus could lead to enhanced handoff efficiency. Evaluating the efficacy of MD scheduling to enhance continuity is currently subjective.

**Methods:**

An MD group consisting of 9 attending physicians and 7 fellows redesigned its pediatric intensive care unit (PICU) coverage schedule with the goal of enhancing continuity of care. The attending PICU MDs were formally surveyed to rate the impact of the schedule change on continuity and efficiency (5 point Likert scale: 1 = worse, 3 = no change, 5 = better). A Handoff Continuity Score (HCS) was developed and used to analyze the 30-bed PICU MD schedule for continuity and handoff efficiency. MD service and call schedules were evaluated for 6-month periods before and after the schedule redesign. The HCS for each schedule was calculated by considering every shift change, or handoff, in the scheduling horizon, and assigning scores to oncoming physicians based on previous days worked. Specifically, for each handoff, each oncoming MD receives a score between 0 and 1, calculated as the summation of a series of 'familiarity factors', one for each recent day worked. The scores for all oncoming MDs are averaged to determine the score for that specific handoff, and the HCS is the average of all handoff scores. The HCS was incorporated into an integer programming (IP) model for scheduling MDs to maximize continuity. A z-test was used to assess the significance of improvement in the HCS.

**Results:**

The HCS before and after redesign was 0.57 and 0.68, respectively (19% increase, p < 0.01). Mean MD rating was 4.22 ± 0.56 for continuity, and 4.00 ± 0.65 for efficiency. With the goal of further improving the HCS and (partly) automating and streamlining the scheduling process, the IP was developed to populate physician service and night-call schedules while conforming to scheduling constraints; IP-generated schedules improved the HCS to 0.79 (39% increase).

**Conclusions:**

The increased HCS was associated with the MD qualitative assessment of enhanced continuity and efficiency after implanting a schedule change. The IP identified the potential for additional scheduling improvements.

## Introduction

Continuity of care improves patient satisfaction [1], whereas the fragmentation of care may have a negative impact on patient outcomes. Epstein and colleagues [2] found a statistically significant relationship between fragmentation of patient care and length of stay (LOS) among patients admitted for pneumonia or heart failure. As staffing models for inpatient hospital settings transition to shift-work because of increased duty hour restrictions and life-style choices by physicians (MDs), serious concerns arise regarding the resulting lack of continuity for patients and MDs and potential impacts on quality of care and education of residents [3].

Fragmented care leads to increased handoffs, or the transfer of patient care from one MD to another. Communication between MDs during handoff of inpatients is a 'vital link in the continuity of patient care' [4] but may be insufficient to relay all pertinent information, and 'communication failures have become widely recognized as a leading safety hazard in health care' [5]. On the basis of a review of controlled studies of interventions, Arora and colleagues [6] provide preliminary recommendations for improving handoffs, but further studies are needed to identify best practices [7,8].

It is intuitive that an MD's familiarity with a patient prior to starting work has the potential to make the handoff process more efficient. Few studies investigate the benefits of familiarity or continuity during MD-to-MD handoff of inpatients, and improving scheduling models to enhance continuity and familiarity could provide a tool to help with handoff efficiency and quality. Evaluating the efficacy of MD scheduling to provide continuity is currently subjective.

We propose, as one method for improving patient and MD continuity, a Handoff Continuity Score (HCS) as a novel approach for objectively assessing continuity and familiarity with various scheduling models. We use the HCS to analyze MD duty schedules before and after the implementation of a schedule structure redesign intended to maximize patient and MD familiarity and continuity within the context of defined scheduling constraints. We incorporate the HCS into a mathematical model developed to automate and improve the process of MD duty schedule generation. The purposes of this study are (a) to develop the HCS as an objective measure of MD and patient continuity, (b) to validate the HCS and its correlation to MD perceptions of continuity and familiarity within a small group of MDs, (c) to use the HCS to measure improvement in continuity following an MD duty schedule redesign, and (d) to assess the application of a mathematical model for constructing MD duty schedules that optimize the HCS.

## Materials and methods

An MD group, consisting of nine pediatric critical care medicine (PCCM) attending MDs and seven PCCM fellows, redesigned its pediatric intensive care unit (PICU) MD coverage schedule to enhance continuity. The PICU is a 30-bed multidisciplinary medical-surgical quaternary care unit and provides care for acutely ill patients, including those receiving liver transplants, continuous renal replacement therapies, or extracorporeal life support. The weekly service schedule for the unit consists of two teams (A and B), each including one attending MD and one fellow providing daytime coverage. The teams treat two separate groups of patients, but MDs from team A are somewhat familiar with the patients treated by team B, and vice versa, because of proximity, collegiality, early-morning fellow sign-outs (informal), and formal sign-outs that include both teams and the oncoming night call MDs. Night call shifts are covered by one attending MD and one fellow.

Prior to the redesign of the schedule, the attending MDs would take service for 1 week (only the 5 consecutive weekdays), which we call a 'service block'. Night call and weekend coverage could be filled randomly by on-service or off-service MDs. The fellows took service for 7 days at a time, and on-service fellows took call 1 or 2 nights per week. An off-service fellow took the remaining night call shifts. During the redesign, the schedule was changed to have the attending MDs take service for 7 days in a row and the fellows for overlapping 14-day periods, in which a new fellow would start a service block every Monday. This ensures that, each week, a fellow who was on service the previous week is on service. The night call coverage schedule was redesigned as shown in Table 1 to provide patient-MD familiarity overnight. Both pre- and post-redesign schedules were constructed manually by one of the PCCM attending MDs.

**Table 1:** Physician schedule redesign of weekly night-call schedule

	Monday	Tuesday	Wednesday	Thursday	Friday	Saturday	Sunday
Attending on call	MD from team A	Off-service attending	Off-service attending	MD from team B	Off-service attending	Off-service attending	Off-service attending
Fellow on call	Night float fellow	MD from team A	MD from team B	Night float fellow	Night float fellow	MD from team A	MD from team B

MD, physician.

The institutional review board of the Children's Healthcare of Atlanta approved this study and waived the need for informed consent as this was a quality initiative that did not involve individual patients or interventions. We developed a scoring method, based on the following assumption, to effectively capture the continuity at handoff.

### Assumption

The level of familiarity that an MD has with their patients (a) increases with successive (or multiple) on-duty days in which the MD sees the patients and (b) decreases as the number of off-duty days increases (for example, to more than the average LOS of patients). Intuitively, an MD who sees a patient for a number of consecutive days will be more familiar with and informed about the patient's condition. Conversely, an MD who sees a patient after a multi-day break would need some time to become refamiliarized with the patient's progress and current state.

The nine attending MDs were informally surveyed to determine a numerical estimate of continuity at each handoff on the basis of prior days worked for the oncoming MDs in the PICU. For each oncoming MD, a continuity score, $\sum_{i=1}^{N}F_{i}X_{i}$, is calculated on the basis of previous days worked (see the notation in Table 2) by using familiarity factors developed on the basis of results of the survey (Table 3). For this initial analysis, *N *was defined as the average LOS in the PICU (5 days), where 'days' are equivalent to 24-hour periods.

**Table 2:** Notation of variables in continuity score

N	Patient length of stay (5 days in our analysis)
F_i_	A familiarity factor dependent on the length of time since last shift
X_i_	1 if the physician worked between i-1 and i days ago, 0 otherwise

**Table 3:** Familiarity factors

Number of days since previous shift	< 1	1 to < 2	2 to < 3	3 to < 4	4 to < 5
Familiarity factor	0.5	0.25	0.15	0.075	0.025

Each oncoming MD could receive a score of between 0 and 1 (1 = most familiar). The score for the handoff is calculated as the average continuity score over all oncoming MDs. For a complete MD schedule, the HCS is calculated as the average of all handoff scores and ranges from 0 to 1.

As an example, consider the 1-week schedule shown in Table 4. Attending MDs A, B, C, and D and fellows F, G, H, and I are assigned to service (8 a.m. to 4 p.m.) and call (4 p.m. to 8 a.m.) shifts throughout the week. In this schedule, consider the call shift that starts at 4 p.m. on Saturday with MDs D and F. Note that MD F is scheduled to work service every day during the week whereas MD D is scheduled to work the Wednesday call shift, which is 2 to less than 3 days before the start of the Saturday call shift. Thus, the continuity scores for these MDs are calculated as shown in Table 5. The handoff achieves a score of 0.575.

**Table 4:** Sample 1-week schedule of attending physicians and fellows

	Monday	Tuesday	Wednesday	Thursday	Friday	Saturday	Sunday
8 a.m. to 4 p.m.	A, B F, G	A, B F, G	A, B F, G	A, B F, G	A, B F, G	A, B F, G	A, B F, G

4 p.m. to 8 a.m.	A, H	C, F	D, G	B, H	C, I	D, F	C, G

Attending physicians: A, B, C, and D; fellows: F, G, H, and I.

**Table 5:** Sample calculation of Handoff Continuity Score

Number of days since previous shift	< 1	1 to < 2	2 to < 3	3 to < 4	4 to < 5	Sum (continuity score)
Physician D	-	-	0.15	-	-	0.15
Physician F	0.5	0.25	0.15	0.075	0.025	1
The handoff score is the average of the physician continuity scores →						(0.15+1)/2 = 0.575

To compare the HCS before and after the schedule redesign, we evaluated MD service and night call schedules for 6-month periods. A z-test was used to assess significance of improvement in the HCS. MDs were asked to rate the impact of the schedule change on continuity and efficiency (see Additional file 1 for survey questions).

To (partially) automate and improve the process of schedule generation, we developed an integer program (IP), which is a mathematical model (a system of equations and inequalities) consisting of an objective function and constraints [9]. The constraints ensure that the requirements of the Accreditation Council for Graduate Medical Education (ACGME) [10] and staffing requirements for the unit (for example, two attending MDs and two fellows on duty for each daytime shift and one attending and one fellow on duty for each night call shift) are not violated. The IP also incorporates soft constraints to accommodate MD requests for time off and the preferred service schedule structure. The soft constraints may be violated only if it is necessary to construct a feasible schedule (and not for the purposes of improving the HCS). The IP was used to construct an MD schedule which maximized the HCS while minimizing violations of the soft constraints. This IP-generated schedule HCS was compared to that achieved by the pre- and post-redesign schedules." For additional details on the IP, see Smalley and colleagues [11].

While the current service schedule structure for the PICU (7-day blocks for attending MDs and 14-day blocks for fellows) was designed with continuity in mind, a better structure with respect to patient and MD continuity may be possible. Using different versions of the IP, we constructed schedules that follow four alternatives to the current service schedule structure and computed the corresponding HCS for each. The five total service schedule structures that we investigated are (1) 7-day blocks for attending MDs and overlapping 14-day blocks for fellows (current schedule structure), (2) overlapping 7-day blocks for attending MDs (in which a new attending MD starts a 7-day service block every Monday and Friday) and overlapping 14-day blocks for fellows, (3) overlapping 4-day blocks for attending MDs (in which a new attending MD starts a 4-day service block every two days) and overlapping 14-day blocks for fellows, (4) overlapping 14-day blocks for fellows but no predefined service block structure for attending MDs, and (5) no predefined service block structure for attending MDs or fellows. Structure 1 is the current service schedule structure in the PICU. Structures 2 and 3 correspond to service schedule structures that MDs in the PICU proposed as potential improvements. We include structures 4 and 5 to determine the best achievable HCS when there is no predefined service block structure (that is, when there is no requirement that MDs be on service for a certain number of multiple consecutive days).

## Results

Eight of the nine attending MDs responded to the survey regarding the impact of schedule redesign on handoff efficiency and continuity. The results (Table 6) show that the MDs note improvement in both handoff efficiency and schedule redesign. Table 7 reports the HCS for pre- and post-redesign schedules as well as the schedule generated by the IP, in which the objective was to maximize the HCS for both service and night call shifts over the scheduling horizon while minimizing violations of soft constraints. We also report the HCS by time of day and MD type. Note that the schedule generated by the IP corresponds to the same time period as the pre-redesign schedule and incorporates the same requests from MDs for time off. Higher scores imply greater familiarity. Given that the HCS is designed to measure continuity and familiarity, we also report the number of call shifts in the scheduling horizon without an on-service MD staffed as it is intuitive that scheduling an on-service MD to the call shift would add continuity at night. The night call familiarity was of particular concern for this MD group for their particular PICU. The descriptions and scores for the alternative service schedule structures we used for comparison are reported in Table 8. For each structure, we incorporated the MD requests for time off which correspond to those in the post-redesign schedule.

**Table 6:** Physician survey results

Physician rating for impact on continuity and handoff efficiency because of schedule redesign	
Continuity	Efficiency
4.22 ± 0.56	4.00 ± 0.65

Data are presented as mean ± standard deviation. Five-point Likert scale: 1 = worse, 3 = no change, and 5 = better.

**Table 7:** Handoff Continuity Score results^a^

	Pre-redesign	Post-redesign		Integer program	
		Score	**Percentage increase**^**b**^	Score	**Percentage increase**^**b**^
HCS of all shifts	0.57	0.68	19% (*P *= 0.0011)	0.79	39% (*P *< 0.001)
HCS of service shifts only	0.64	0.76	19% (*P *= 0.0244)	0.78	22% (*P *= 0.0104)
HCS of night call shifts only	0.50	0.61	22% (*P *= 0.008)	0.80	60% (*P *< 0.001)
HCS of attending physicians only	0.52	0.67	29% (*P *< 0.001)	0.84	62% (*P *< 0.001)
HCS of fellows only	0.62	0.70	13% (*P *< 0.001)	0.74	19% (*P *< 0.001)
Night call shifts without an on-service physician (*n *= 175)	30% (52)	4.6% (8)		3.4% (6)	

^a^Metrics reported for schedules covering 6 months. ^b^Percentage increase over pre-redesign schedule. HCS, Handoff Continuity Score.

**Table 8:** Handoff Continuity Scores for alternative service schedules generated by the integer program

Service schedule structure and description		HCS	HCS of attending physicians only	HCS of fellows only	Night call shifts without an on-service physician
1	7-day blocks for attending physicians and overlapping 14-day blocks for fellows	0.79	0.85	0.74	8
2	Overlapping 7-day blocks for attending physicians (Mon.-Sun. or Fri.-Thurs.) and overlapping 14-day blocks for fellows	0.79	0.84	0.73	19
3	Overlapping 4-day blocks for attending physicians and overlapping 14-day blocks for fellows	0.73	0.73	0.73	1
4	Overlapping 14-day blocks for fellows but no predefined service block structure for attending physicians	0.83	0.93	0.73	21
5	No predefined service block structure for attending physicians or fellows	0.85	0.92	0.77	16

HCS, Handoff Continuity Score.

## Discussion

On the basis of survey results, the attending MDs found that continuity and handoff efficiency were improved by redesigning the on-service and night call shift schedule structure. This was positively correlated with the HCS numerical estimate of familiarity and continuity.

There was a statistically significant increase in the HCS after the schedule redesign. When viewing the HCS by time of day, we see that the HCS for night call shifts is considerably less than the HCS for service day shifts for both pre- and post-redesign schedules, an expected result given the service schedule structures in both. The greatest increase (29%) in the post-redesign HCS is associated with the schedule for attending MDs. The HCS for fellows increased by only 13%. The fellows' schedule is structured to provide concentrated clinical time (a key component for the HCS) in order to provide protected research time during their training. Increasing from 7-day service blocks, which exceeds the PICU LOS (5 days), to 14-day service blocks resulted in only a modest improvement to the HCS. In addition, ACGME duty hour restrictions provide a rigorous hard constraint that may have limited the improvement in the HCS.

While the schedule redesign improved the HCS, the IP identified the potential for additional scheduling improvements. However, the best HCS was achieved by a schedule that required attending MDs who are on service during a given week to work alternating call shifts that week, without any other attending MDs scheduled. Note the resulting much-improved HCS for night call shifts for the IP-generated schedule over the pre- and post-redesign schedules. There is only a modest improvement in the HCS for service day shifts over the post-redesign schedule because this score is determined largely by the service block structure implemented post-redesign.

The post-redesign schedule ensures that at least six night call shifts per week have an on-service MD (attending MD or fellow) scheduled. Table 7 indicates that, post-redesign, only 8 night call shifts in the 6-month schedule had no on-service MD scheduled, a decrease of 85% from the 52 pre-redesign. The overall HCS increased by 19% post-redesign. The IP-generated schedule did not greatly reduce (in comparison with the post-redesign schedule) the number of night call shifts without an on-service MD (6 versus 8), but the overall HCS increased by 16% over the post-redesign schedule. Thus, there is not a linear relationship between the HCS and the number of night call shifts without an on-service MD. Other factors impact the HCS such as daytime continuity and familiarity among off-service MDs assigned to night call shifts.

While the IP-generated schedule maximizes continuity, its implementation may lead to fatigue and negatively impact quality of care. In cases like this, adjustments can be made in two ways. First, additional constraints can be added to the IP to prevent unfavorable scheduling situations and to address MD preferences (for example, to set a limit on the number of night call shifts per week) to generate a schedule that maximizes continuity without relying on manual generation of schedules. The alternative is that the IP-generated schedule can be manually manipulated and the HCS recalculated to determine the impact of changes on continuity. Given that MD schedules often are made well in advance, changes are needed from time to time to adjust for new requests from MDs and other unavoidable situations. The HCS provides some objective assessment of the impact of such changes on continuity of patient care as MDs evaluate alternatives.

The mathematical model we developed is not the first for scheduling MDs in a hospital setting. Sherali and colleagues [12] addressed the problem of scheduling medical residents to night shifts over the course of 4 to 5 weeks by using a mixed-integer program and heuristics that exploit the network structure of the problem. Topaloglu [13,14] used goal programming to assign emergency medicine residents to day and night shifts over a 1-month planning horizon and applied sequential and weighted methods for solving an optimization model with multiple objectives to the problem of assigning residents to on-call shifts based on levels of seniority. Ovchinnikov and Milner [15] developed a user-friendly spreadsheet model for assigning first- to fourth-year residents to on-call and emergency rotation shifts in a radiology department over a 1-year period, and Cohn and colleagues [16] addressed the problem of assigning 10 to 20 residents to five types of on-call shifts in three distinct hospitals over a 1-year period by solving multiple nested IP formulations to address the different metrics. Our IP has some similarities with previous work but, to the best of our knowledge, is the first model designed to address the impact of MD scheduling on MD and patient continuity.

The HCS gives consideration to various schedule structures to be tested with an objective quantitative assessment of the impact on continuity. In comparing the scores reported for the various service schedule structures applied to the PICU, we see that the greatest HCS can be achieved by having no predefined service block structure for attending MDs or fellows. The version of the IP used for this schedule structure has fewer constraints than others (that is, no predefined service block length for attending MDs or fellows or both), and therefore we would expect this alternative structure to achieve the highest HCS. Removing predefined service block length constraints, the IP can generate an optimal MD duty schedule with respect to continuity. However, in this case, the schedule generated with this structure requires that attending MDs and fellows be on service for several weeks to a month at a time and may not adhere to MD preferences or be feasible. The number of call shifts without an on-service MD is minimized by structure 3, but the corresponding HCS is also the lowest of the service schedule structures that we tested. The HCS and number of night call shifts without an on-service MD are useful assessments that MD groups can use to tailor a service and call schedule to the needs of their unit or MDs or both. The current service schedule structure (structure 1 in Table 8) is preferred among MDs in the study PICU because, among candidate schedules with the 7-day preferred service structure for attending MDs, it has the fewest night call shifts without an on-service MD associated with the highest HCS.

Familiarity among oncoming MDs has the potential to improve the handoff process itself. However, familiarity alone does not reduce the need for careful discussion during the handoff of a patient's care from one MD to another. Owing to the dynamic nature of illnesses, a patient's condition evolves over time. New information may become available and the course of treatment may change from one day to the next. Furthermore, factors such as new admissions, discharges, bed occupancy, and MD fatigue impact handoff efficiency. Future research opportunities include developing a fatigue factor for assessing the impact of scheduling changes on continuity and fatigue as it impacts the handoff process.

We did not take into account Hawthorne effects among the nonblinded participating MDs. As the first application of this approach, the analysis above relies on the perceptions of an MD group within one PICU. Furthermore, only attending MDs were surveyed and therefore we can make no assumptions regarding the perceived improvement (if any) felt by fellows in this MD group following the schedule redesign. A larger sample of MDs is needed to truly validate the HCS. The potential far-reaching implications of the HCS, particularly as it might impact patient outcomes, has not been studied and is beyond the scope of this article.

## Conclusions

To maintain good quality of care, it is important that MDs be familiar with their current patients; familiarity contributes to MD and patient continuity. MD familiarity and continuity are tools that can be used to help improve efficiency and reduce miscommunications that can occur during the handoff process. The proposed HCS is a novel approach for objectively assessing continuity and familiarity given our intuitive assumptions. The results of the MD survey reveal that the HCS is associated with perceived improvement in continuity and handoff efficiency from the schedule redesign. With a mathematical model, an MD schedule can be optimized with respect to the HCS with the goal of maximizing continuity. The IP and HCS together facilitate resource optimization, particularly in the face of increased duty hour constraints. While this initial study assessing an objective scoring method for measuring MD and patient continuity is limited to a small group of MDs, implications may be far-reaching, and further study is needed to assess the applicability of such an approach to other units and institutions.

## Key messages

• A proposed Handoff Continuity Score correlates with physicians' perceptions of enhanced continuity and patient familiarity.

• An objective scoring method can be used to analyze a physician duty schedule for physician and patient continuity.

• With a mathematical model, a physician schedule can be optimized with respect to continuity.

## Abbreviations

ACGME: Accreditation Council for Graduate Medical Education; HCS: Handoff Continuity Score; IP: integer program; LOS: length of stay; MD: physician; PCCM: pediatric critical care medicine; PICU: pediatric intensive care unit.

## Competing interests

The authors declare that they have no competing interests.

## Authors' contributions

This was a collaborative study and all three authors were closely involved in all aspects of the study. The lead writer was HKS, and PK and AV closely supervised/mentored. All authors read and approved the final manuscript.

## Supplementary Material

Additional file 1 **Physician Survey Questions and Results**. Questions and results from survey of attending physicians, scored on a Likert Scale. Click here for file
