# Supplementary material for: Development of a handoff continuity score to improve pediatric ICU physician schedule design for enhanced physician and patient continuity
Source: Crit Care. 2011 Oct 21;15(5):R246. doi: 10.1186/cc10504 (PMC3334797; doi:10.1186/cc10504)
Supplement: Additional file 1 — Physician Survey Questions and Results. Questions and results from survey of attending physicians, scored on a Likert Scale. [file cc10504-S1.PDF]

Additional Data File 1 - Physician Survey Questions and Results

	Mean	Standard Deviation
**How has the new schedule impacted continuity of care during night-call coverage?**	**4.25**	**0.49**
**Has the familiarity helped with handoff efficiency during sign-out?**	**4.00**	**0.58**
**How has the new schedule helped with weekend continuity?**	**4.13**	**0.69**
**Has the familiarity helped with rounding efficiency on the weekends?**	**3.88**	**0.82**
**How has the schedule impacted fatigue?**	**2.38**	**1.13**
**Is the enhanced continuity worth the increased fatigue?**	**3.75**	**1.35**
5 point Likert scale, 1 = worse, 3 = no change, 5 = better		
